# Enhancing researcher capacity to engage youth in research: Researchers’ engagement experiences, barriers and capacity development priorities

**DOI:** 10.1111/hex.13032

**Published:** 2020-03-14

**Authors:** Lisa D. Hawke, Karleigh Darnay, Jacqueline Relihan, Mohammad Khaleghi‐Moghaddam, Skye Barbic, Lisa Lachance, Shelly Ben‐David, Marion Brown, Srividya Iyer, Gloria Chaim, Sophie Soklaridis, Sean A. Kidd, Tanya Halsall, Steve Mathias, Joanna Henderson

**Affiliations:** ^1^ Centre for Addiction and Mental Health; ^2^ University of Toronto; ^3^ Foundry; ^4^ University of British Columbia; ^5^ Centre for Health Evaluation Outcome Sciences; ^6^ Wisdom2Action; ^7^ Dalhousie University; ^8^ UBC Okanagan; ^9^ Department of psychiatry, McGill University; ^10^ ACCESS Open Minds; ^11^ Douglas Hospital Research Centre; ^12^ University of Ottawa; ^13^ The Royal's Institute of Mental Health Research, affiliated with the University of Ottawa

**Keywords:** capacity development, patient engagement, youth, youth‐adult partnerships

## Abstract

**Background:**

There is increasing emphasis on engaging youth in research about youth, their needs, experiences and preferences, notably in health services research. By engaging youth as full partners, research becomes more feasible and relevant, and the validity and richness of findings are enhanced. Consequently, researchers need guidance in engaging youth effectively. This study examines the experiences, needs and knowledge gaps of researchers.

**Methods:**

Eighty‐four researchers interested in youth engagement training were recruited via snowball sampling. They completed a survey regarding their youth engagement experiences, attitudes, perceived barriers and capacity development needs. Data were analysed descriptively, and comparisons were made based on current engagement experience.

**Results:**

Participants across career stages and disciplines expressed an interest in increased capacity development for youth engagement. They had positive attitudes about the importance and value of youth engagement, but found it to be complex. Participants reported requiring practical guidance to develop their youth engagement practices and interest in a network of youth‐engaged researchers and on‐going training. Those currently engaging youth were more likely to report the need for greater appreciation of youth engagement by funders and institutions.

**Conclusions:**

Engaging youth in research has substantial benefits. However, skills in collaborating with youth to design, conduct and implement research have to be learned. Researchers need concrete training and networking opportunities to develop and maximize these skills. They also need mechanisms that formally acknowledge the value of engagement. Researchers and those promoting youth engagement in research are encouraged to consider these findings in their promotion and training endeavours.

## 1 INTRODUCTION

Researchers and funding bodies increasingly consider it essential to engage service users and individuals with lived experience across the disciplines in research relevant to them, in order to improve research quality and relevance.^1^, ^2^, ^3^, ^4^ Canada's Strategy for Patient‐Oriented Research emphasizes the critical importance of engaging patients in health services research, as well as the need for building capacity in this way of working.^5^ The movement towards patient engagement is also reflected in frameworks in other countries, such as the Patient‐Centered Outcomes Research Network^6^ in the United States and the National Institute for Health Research INVOLVE framework^7^ in the United Kingdom. Engaging individuals with lived experience of the health issue under investigation can lead to more relevant research questions, more feasible processes and stronger research uptake; it also helps bridge the knowledge‐to‐practice gap.^4^

Engaging youth in research is valuable across the disciplines, including research on issues of health and well‐being,^2^ health promotion^8^ and mental health,^9^ and also issues of social inequity,^10^ community development,^11^, ^12^ organizational change^13^ and educational reform.^14^ While traditionally youth have been participants in research projects, youth engagement models call for youth to be full partners in the research process.^3^, ^15^ Youth engagement is particularly important in the mental health and substance use services domain.^1^, ^2^, ^16^ By engaging youth in collaborative research activities, research is more likely to be aligned with the needs and priorities of young people; it thereby becomes more likely to be feasible, easily adopted and implemented, while producing results that are sustained over time.^3^, ^11^ However, like any other skill, collaborating with youth to develop, design, conduct and implement research has to be taught and learned; this is essential in order to guide researchers in engaging authentically, avoiding tokenism and ensuring the safety of the youth.^15^, ^17^

A recent study examined the attitudes and engagement experiences of early career researchers regarding the engagement of youth and adults in mental health research.^16^ That study showed positive attitudes towards engagement among researchers, but also several barriers, such as challenges to recruiting, a lack of a supportive institutional and broader community environment, and limited practical resources. They also found that engagement was more common with adults as compared to youth. That study highlights the need for further work to understand the engagement experiences, attitudes and barriers in the youth sector across career stages with a view to enhancing training and capacity development.

**Figure 1:**
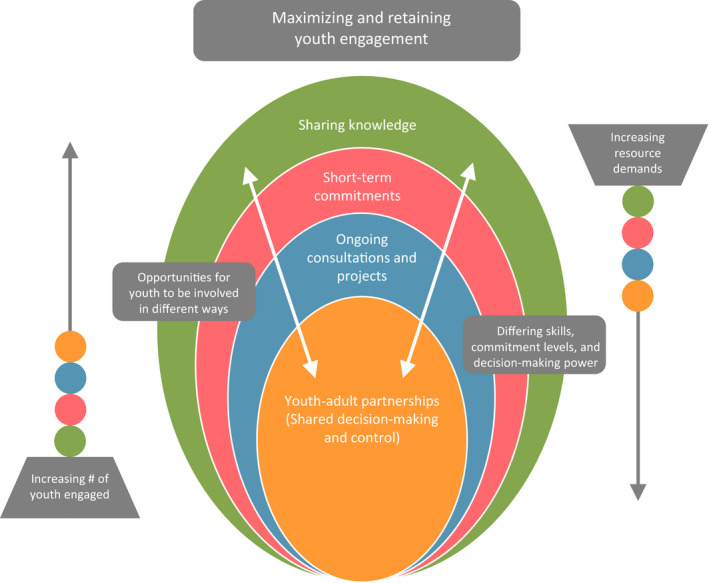
McCain Model of Youth Engagement. Figure licensed under CC‐BY, as presented in Heffernan et al^15^

Reflecting a commitment to youth‐engaged research, the leaders of the Margaret and Wallace McCain Centre for Child, Youth & Family Mental Health co‐developed the McCain Model of Youth Engagement together with youth with lived experience of mental health service use who were on staff as youth experts (see Heffernan et al,^15^ for details). The McCain Model of Youth Engagement (Figure 1) describes various levels of youth engagement that can provide young people with a range of opportunities that reflect differences in youth capacity, commitment and availability. This makes it possible to ensure that diverse youth voices are leveraged to inform research processes. Different levels of engagement also allow for flexibility to support various types of engagement within research, noting that different projects may be more suited for higher or lower levels of engagement. The McCain Model also emphasizes the importance of authentically valuing youth expertise and creating opportunities for meaningful participation. From this work, the team outlined practical guidelines for researchers, with the goal of developing a comprehensive curriculum to help researchers engage youth effectively.^17^ We have also showcased some of the impacts that youth engagement has had on research conducted from a patient‐oriented research framework.^18^

Leveraging the team's expanding experience in and commitment to engaging youth in research, we collaborated with pan‐Canadian partners, including youth, on the INNOVATE Research project. Through this project, a national team developed and presented a thorough youth engagement curriculum; the curriculum aims to build researcher capacity to engage youth in meaningful, authentic ways in all aspects of a research project, from design and development through to knowledge translation.^19^ The team also assessed researchers’ youth engagement practices, attitudes and capacity development needs.

### 1.1 Objective

The current study aims to understand the profiles, youth engagement capacity development needs and barriers of researchers with and without youth engagement experience. By enhancing our understanding of the profiles of researchers interested in youth engagement training initiatives, it will be possible to tailor training initiatives to their learning needs, while also addressing barriers to increase capacity in youth engagement in research.

## 2 METHOD

### 2.1 The INNOVATE research project

The INNOVATE Research team is a pan‐Canadian team of youth‐engaged researchers and youth from the Centre for Addiction and Mental Health, University of Toronto, McGill University, Dalhousie University, the University of British Columbia, the University of Ottawa, Wisdom2Action, Foundry, Frayme, ACCESS Open Minds and the Douglas Hospital Research Centre. The project team has co‐developed—with youth—a thorough youth engagement curriculum for researchers, which the team used to co‐deliver workshops and coaching sessions to teams of researchers in Toronto, Halifax and Vancouver, Canada, as well as a national webinar, teaching the fundamentals of youth engagement. This study presents the data collected from prospective workshop attendees prior to the workshops.

### 2.2 Participants and procedure

The sample consists of N = 84 individuals who expressed interest in registering for a 1‐day INNOVATE Research workshop in Halifax, Toronto or Vancouver, Canada, and who completed the pre‐workshop survey. The sampling goal was 20‐30 participants per workshop, for a feasible interactive workshop experience. Participants were recruited by circulating a workshop flyer through the research team's networks and to area universities; the flyer was circulated electronically to researchers and key contacts in academic settings in each location, with passive snowball sampling as the flyer was further circulated by recipients and those interested were invited to contact the project team. The workshop was advertised as a full‐day event with learning goals including: (a) helping attendees adopt a stakeholder‐informed research approach in line with emerging funding priorities; (b) learning how youth engagement can improve research validity, relevance and impact; (c) the opportunity to work with leading research to plan for youth engagement in a project; and (d) availability of post‐workshop coaching sessions. To be included in the current sample, the participant must have contacted the research team about attending the INNOVATE Research workshop in one of the three locations, expressed willingness to complete the pre‐workshop survey and provided their contact information to receive the survey. They must then have followed the web link to provide informed consent and answer the pre‐workshop survey. They were not required to follow through to register for and participate in the workshop to be included in the sample; however, 84.5% of study participants did ultimately attend the workshop (N = 71). There was no difference in current youth engagement experience between those who ultimately attended the workshop and those who did not in terms of age, sex or engagement experience. Completion of the questionnaires entitled the participant to a $20 discount (50%) on attending the workshop. One individual consented, but did not complete the questionnaire set, and was therefore removed from the sample. Research Ethics Board approval was obtained from the Centre for Addiction and Mental Health; ethics approval was also obtained from partner universities (University of Toronto, Dalhousie University, University of British Columbia).

### 2.3 Measures

A series of measures was hosted on REDCap electronic data capture system^20^ and administered electronically. The online survey included descriptive information about sociodemographic and professional profiles, as well as current practices and perceived barriers to youth engagement in research. These items were developed collaboratively among our pan‐Canadian team through an iterative feedback and refinement process and included issues such as practical engagement practices, human and financial resources, relevance and ethical issues. As a pilot stage, we collected information from 83 researchers in our network regarding barriers to youth engagement and learning needs, to help shape the final survey; these researchers were not study participants, but rather colleagues who helped to refine the data collection tool.

Also included in the survey was the Service Provider Adopter and Innovation Characteristics Questionnaire (SPAICQ),^21^, ^22^ a 21‐item scale used in implementation science. The SPAICQ consists of standard question stems, which are adapted to the construct of interest, in this case, the engagement of youth in research. The SPAICQ contains subscales examining adopter characteristics and innovation characteristics of a given construct, that is, concern (four items, eg ‘I believe engaging youth in research is important’), self‐efficacy (four items, eg ‘I can engage youth effectively in research’), complexity (five items, eg ‘Strategies for engaging youth in research are easy to implement’), compatibility (four items, eg ‘Engaging youth in research fits in well with my organization’) and relative advantage (four items, eg ‘Engaging youth in research improves the overall quality of research’). For each subscale, an average score on a 1‐5 scale is used. The Level of Use (LOU) questionnaire was also administered.^23^ The LOU categorizes the use of an innovation from 0 (non‐use) to 4 (high use). It is a 22‐item scale consisting of standard stems that are being adapted to insert the innovation being assessed, that is, youth engagement in research. Categorical results reflect the level of innovation use with the highest average score for that individual participant.

### 2.4 Analyses

Data were analysed descriptively to understand the characteristics and engagement practices of researchers interested in developing their youth engagement skills, as well as their perceived barriers to engagement and areas of need for engagement capacity building. Chi‐square tests were used to compare results among participants who reported that they currently engage youth in research vs those who do not on categorical variables, with Fisher's exact tests when cell sizes were small. The self‐reported single‐item indication of current engagement experience (yes, no) was used for group comparisons; the single dichotomous item was significantly associated with the five categories in the LOU scale (*P* < .001). Independent sample *t* tests were used to compare the two groups (currently engage youth vs not) on the SPAICQ subscales. A significance threshold of alpha <0.05 was retained. SPSS version 24 was used.^24^

## 3 RESULTS

Participant characteristics are presented in Table 1. The sample represents a diversity of ages, disciplines and levels of education and research experience. The majority of participants were female, under the age of 40, and with five years or less of research experience; nearly half of participants (45%) spent more than half of their time on research.

**Table 1:** Sociodemographic characteristics and professional profiles of participants

Characteristic	N (%)
Age	
20‐29	35 (41.7%)
30‐39	27 (32.1%)
40‐49	14 (16.7%)
50+	8 (9.5%)
Sex	
Male	10 (11.9%)
Female	73 (86.9%)
Other	1 (1.2%)
Primary position	
University professor/administrator	15 (17.9%)
Community/hospital‐based researcher	17 (20.2%)
Trainee (PDF, PHD, other, student)	29 (34.5%)
Research staff	16 (19.0%)
Other	5 (6.0%)
Education	
Bachelor's or less	27 (32.1%)
Master's	29 (34.5%)
PhD, MD	27 (32.1%)
Primary discipline	
Psychology	27 (32.1%)
Social work	19 (22.6%)
Sociology	13 (15.5%)
Medicine (psychiatry or other)	11 (13.1%)
Other health	16 (19.0%)
Other social sciences	18 (21.4%)
Other	8 (9.5%)
Years of experience in youth‐relevant issues	
Less than 1 y	17 (20.2%)
1‐5 y	41 (48.8%)
6‐10 y	15 (17.9%)
11+	10 (11.9%)
Percentage of time spent on research	
0%	4 (4.8%)
1%‐25%	26 (31.0%)
26%‐50%	16 (19.0%)
51%+	38 (45.2%)

**Figure 2:**
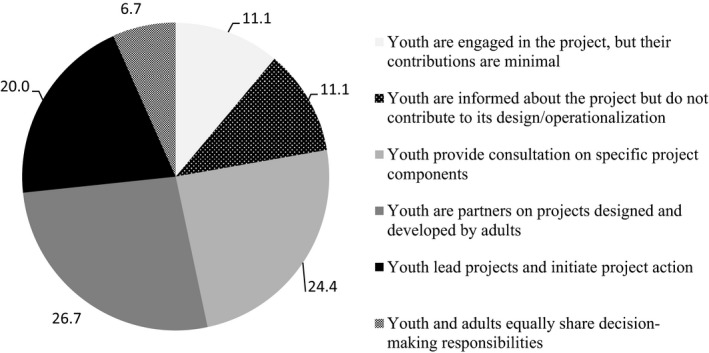
Extent of youth engagement among participants who reported currently engaging youth in research (per cent)

The engagement experience of the participants is presented in Table 2 and Figure 2. The vast majority (85.7%) of participants considered themselves very familiar or somewhat familiar with stakeholder‐engaged research; familiarity with stakeholder‐engaged research did not differ by level of education (χ^2^(4) = 3.468, *P* = .483), although higher familiarity was expressed by participants who reported that they currently engage youth in research (χ^2^(2) = 12.071, *P* = .002). Over half of the sample (53.6%) reported currently engaging youth in research, and two‐thirds (66.7%) reported that they conduct stakeholder‐engaged research. Consultation on project components and partnership on adult‐designed project represented the majority of engagement practices (Figure 2). The most common types of activities in which the participants engage youth in research were the research design stage (44.0%) and the planning of knowledge translation activities (42.9%). The least common areas of engagement were co‐authoring manuscripts (10.7%), co‐presenting at conferences (22.6%) and initial research planning (22.6%).

**Table 2:** Current youth engagement profiles of participants

Characteristic	N (%)
Familiarity with engaged research	
Very familiar	29 (34.5%)
Somewhat familiar	43 (51.2%)
Not very familiar	12 (14.3%)
Currently do stakeholder engage research	
No	28 (33.3%)
Yes	56 (66.7%)
Currently engage youth	
No	39 (46.4%)
Yes	45 (53.6%)
Level of use of youth engagement	
Non‐use	17 (20.2%)
Very low use	21 (25.0%)
Low use	27 (32.1%)
Moderate use	6 (7.1%)
High use	13 (15.5%)
Number of projects that include youth engagement	
0	32 (38.1%)
1	31 (36.9%)
2+	20 (23.8%)
How youth are engaged	
Design (methodology, recruitment strategies, measurement selection)	37 (44.0%)
Identification of target audiences and knowledge translation strategies	36 (42.9%)
Co‐analysing/interpreting findings	29 (34.5%)
Co‐developing knowledge translation materials	22 (26.2%)
Initial planning (identify research question, writing grant)	19 (22.6%)
Co‐presenting at conferences	19 (22.6%)
Co‐authoring manuscripts	9 (10.7%)

The SPAICQ results (Table 3) reveal that participants had high levels of concern or appreciation of youth engagement, found it compatible with the way they work and acknowledged the relative advantage of working in this way, regardless of whether they reported currently engaging youth (mean scores of >4.0 on a 1‐5 scale). However, their sense of self‐efficacy in engaging youth in research was more moderate (*M* = 3.437, SD = 0.653), and they had the sense that this was quite a complex process (*M* = 2.938, SD = 0.517). Two key differences emerged between participants who reported that they currently engage youth in research vs those who do not: Those with current engagement experience reported significantly higher levels of compatibility (*t*(82) = 2.353, *P* = .021) and relative advantage (*t*(82) = 2.490, *P* = .015), that is, they found youth engagement to be highly compatible with the types of work they do and considered that it provided meaningful benefits to their research.

**Table 3:** Attitudes towards youth engagement, as a whole and by current youth engagement status

Subscale	Total	Does not engage youth	Engages youth	*t*(82)	*P*
	*M* (SD)	*M* (SD)	*M* (SD)		
Concern	4.535 (0.615)	4.462 (0.543)	4.598 (0.671)	1.016	.313
Self‐efficacy	3.437 (0.653)	3.353 (0.713)	3.509 (0.595)	1.097	.276
Complexity	2.938 (0.517)	2.918 (0.502)	2.956 (0.534)	0.331	.742
Compatibility	4.024 (0.662)	3.846 (0.687)	4.178 (0.604)	2.353	.021
Relative advantage	4.137 (0.733)	3.930 (0.607)	4.317 (0.789)	2.490	.015

Multiple barriers to engaging youth in research were endorsed, as well as areas for capacity development (Tables 4 and 5). Participants endorsed an average of 2.643 barriers (SD = 1.788); the number of barriers endorsed did not differ between those who reported engaging youth and those who did not [*F*(1, 82) = 0.055, *P* = .815]. Frequently endorsed barriers included practical issues regarding the ‘how to’ of youth engagement (ie how to engage youth on a practical level, how to prepare youth for engagement, how to get a representative group of youth together, each endorsed by over 40% of participants). For 10 out of 11 listed barriers, there was no difference between participants who currently engage youth in research and those who do not, although there was a non‐significant trend towards those engaging youth being more likely to report budgetary challenges (χ^2^(1) = 3.020, *P* = .082). Researchers who reported that they did not currently engage youth were significantly more likely to report that this way of working was not relevant to the type of research they do; however, cell sizes were small and this should be interpreted with caution.

**Table 4:** Perceived barriers to engaging youth in research among participants, as a whole and by current engagement status

Barrier	Total	Does not engage youth	Engages youth	χ^2^(1)	*P* a
	N (%)	N (%)	N (%)		
1. Not sure how to engage youth on a practical level	38 (45.2%)	19 (48.7%)	19 (42.2%)	0.356	.551
2. Don't know how to prepare youth to engage in research in this way	37 (44.0%)	17 (43.6%)	20 (44.4%)	0.006	.937
3. Don't know how to get a representative group of youth together	34 (40.5%)	17 (43.6%)	17 (37.8%)	0.293	.588
4. Don't have funding to support this	32 (38.1%)	11 (28.2%)	21 (46.7%)	3.020	.082
5. Unsure about the ethical considerations of engaging youth	28 (33.3%)	15 (38.5%)	13 (28.9%)	0.862	.353
6. Don't have time or human resources to support this	18 (21.4%)	8 (20.5%)	10 (22.2%)	0.036	.849
7. Not sure I can appropriately relate to youth or communicate with them effectively	8 (9.5%)	4 (10.3%)	4 (8.9%)	0.045	1.000
8. Department/university doesn't recognize the value of this type of work	7 (8.3%)	3 (7.7%)	4 (8.9%)	0.039	1.000
9. Not relevant to the type of research I do	4 (4.8%)	4 (10.3%)	0 (0.0%)	4.846	.043
10. Not interested in working in this way	1 (1.2%)	1 (2.6%)	0 (0.0%)	1.168	.464
11. Other institutional barrier	9 (10.7%)	5 (12.8%)	4 (8.9%)	0.338	.727

a Chi‐square for items 1‐6, Fisher's exact test for items 7‐11 due to small cell sizes.

**Table 5:** Youth engagement capacity development needs of participants, as a whole and by current engagement status

	Total	Does not engage youth	Engages youth	χ^2^(1)	*P*
	N (%)	N (%)	N (%)		
Strengthened network of youth‐engaged researcher	61 (72.6%)	28 (71.8%)	33 (73.3%)	0.025	.875
Additional training	58 (69.0%)	26 (66.7%)	32 (71.1%)	0.193	.660
Greater funder appreciation of youth engagement	42 (50.0%)	15 (38.5%)	27 (60.0%)	3.877	.049
On‐going consultation	38 (45.2%)	18 (46.2%)	20 (44.4%)	0.025	.875
Greater institutional appreciation of youth engagement	36 (42.9%)	11 (28.2%)	25 (55.6%)	6.382	.012
Enhanced curriculum	35 (41.7%)	16 (41.0%)	19 (42.2%)	0.012	.912
Online training	28 (33.3%)	11 (28.2%)	17 (37.8%)	0.862	.353

Capacity‐building preferences (Table 5) pointed particularly strongly to the need to develop a network of youth‐engaged researchers (endorsed by 72.6% of participants) and to provide on‐going training in this area (69.0%). There were some differences in the perceived capacity‐building needs between those with current engagement experience and those without: Currently engaging youth in research was associated with significantly higher endorsement of the need for greater appreciation of youth engagement by funders (χ^2^(1) = 3.877, *P* = .049) and institutions (χ^2^(1) = 6.382, *P* = .012).

## 4 DISCUSSION

This study examined the profiles and capacity development needs of individuals interested in attending a workshop on engaging youth in research. Results showed interest in youth engagement across disciplines and across career levels, among individuals with and without current experience engaging youth. Participants had positive attitudes towards youth engagement, but reported that it was a complex process. Perceived barriers to youth engagement revolved largely around practical aspects of the processes, pointing to the importance of focused, concrete training and mentorship opportunities. Participants also expressed interest in establishing a network of youth‐engaged researchers.

There were several findings of interest concerning experience in and perceptions of youth engagement among participating researchers. Those who were currently engaging youth perceived youth engagement to be more valuable than their counterparts without this experience. It is possible that this reflects learning through experience; they may have observed the benefits achieved by engaging youth. This is significant as youth‐adult partnerships require strong adult buy‐in to be successful.^15^, ^19^, ^25^ Alternatively, it may be that researchers who have a greater interest in working in this way have made more progress in incorporating it in their work and were therefore more likely to endorse current engagement experience. Nevertheless, it may be important to raise awareness of how youth engagement can enhance research impacts among researchers who do not engage youth in their work, while giving researchers the opportunity to experience youth engagement to enhance their interest and buy‐in. Those with current experience engaging youth were also more likely to call for more appreciation of youth engagement at the institutional and funder levels, despite few participants endorsing this type of appreciation as a barrier. This may also reflect learning through experience; participants may have observed a lack of funder and institutional buy‐in once they began working in this way; they may also choose to work in a youth‐engaged way despite the lack of institutional buy‐in based on their high level of belief in the importance of this area of work, considering institutional buy‐in not as one of the leading barriers to working in this way.

The ways in which participants reported engaging youth are also of particular note. Project design was the most common stage, with substantially lower levels of engagement in the initial planning stage and presenting the findings in presentation and manuscript writing activities; high levels of full engagement as equal decision makers were rare. Engaging youth at the outset, in identifying research priorities, is of paramount importance to ensure that the research questions asked are relevant to the lived experience of young people.^2^ When engaged throughout a research project, their contributions deserve to be acknowledged, such as in the form of co‐authorship.^17^ Emphasizing engagement throughout a project's life cycle in fulsome ways should be a priority of engagement training activities.

There is increasing emphasis on stakeholder, service user and youth engagement, both among funding bodies^7^, ^26^ and in the academic literature.^4^, ^15^, ^17^, ^27^ Although there is a need for more systematic evaluation of the impact of engagement,^28^ a growing literature is describing the benefits of patient engagement in health research, such as more appropriate research topics and processes, increased recruitment success and a bridged knowledge‐to‐action gap.^2^, ^29^, ^30^ Engaging end users in the research process requires adaptations to the traditional research process and a new way of thinking, with particular considerations when engaging young people. While some literature has been published describing models of ‘youth‐engaged’ or ‘youth‐oriented’ research,^9^, ^15^ there is a paucity of clear documentation available to researchers on the practical steps required to engage youth authentically, across the full lifespan of a research project. Strong leadership is required to engage youth in complex research activities,^9^, ^31^ which requires a champion who can lead the project team in effective engagement, as well as an organizational climate that encourages engagement.

Among participants in the current study interested in increasing their training in this area, it is unsurprising that attitudes towards youth engagement were very positive. Previous work has found positive attitudes towards youth engagement among researchers with diverse levels of experience and knowledge.^27^ Participants in the study, however, also perceived a certain level of complexity to youth engagement and identified the need for more training and networking with other researchers working in this way. Similarly, a qualitative study found that researchers overwhelmingly expressed a wish for more training in youth engagement, with teachings coming specifically from academic perspectives.^27^ That study found that researchers valued youth engagement but felt that it fit more strongly with qualitative research work as opposed to quantitative, and particularly not randomized controlled trials, and was not fully respected by other researchers. A systematic review of patient engagement also found that qualitative research was the most common methodology in which patients are engaged, although findings suggested that engagement is feasible in a wide variety of study designs.^32^ The INNOVATE Research project responds to researchers’ expressed needs by providing academic examples of youth engagement and features a randomized controlled trial as a primary example of successful engagement,^18^ as well as other methodologies. As researchers, institutions and funding bodies continue to advocate for more engagement, they are also encouraged to consider providing concrete training opportunities tailored to researchers, to help them engage youth effectively in a wide variety of research activities and designs. It should be noted that meaningful engagement of youth and adults alike takes time and funding.^27^, ^32^, ^33^ It is a process that requires reflection and, often, greater time to engage young people in a meaningful way.^34^, ^35^ Slower processes may reduce the pace of work in ways that interfere with achieving traditionally valued markers of success, for example, rapid turnaround of traditional benchmarks such as peer‐reviewed manuscripts, grant application submissions on tight deadlines and project operationalization. Participants in the current study who reported engaging youth reported a need for increased appreciation of youth engagement by institutions and funding bodies. This may reflect challenges they have faced in this regard. Given the benefits of youth engagement, we believe this time is worth the adaptations to processes and timelines. We therefore call on academic institutions to formally recognize the value of this way of working and make allowances for the time commitment involved. A supportive academic environment that promotes youth engagement in research has been considered in the literature to increase researchers’ positive attitudes regarding youth engagement.^27^ To enhance this support, institutions might consider adding mandatory reporting on level of engagement as part of regular academic reporting activities, with particular value attributed to projects and teams with higher levels of engagement. Funding bodies and even research ethics boards might consider systematically asking applicants to explicitly describe how they plan to engage service users, including youth, in their projects, with a requirement that engagement activities be described in the project's submission to support the relevance of the research questions asked,^2^ as well as ethical design^2^, ^36^; there could also be a requirement to describe engagement activities in the final report as a measure of accountability. In addition, they may wish to request a description of related impacts, such as a narrative report of the new insights derived from the perspectives of youth engaged on the study. In doing so, they would provide greater incentive and recognition of authentic engagement activities that strengthen the feasibility and relevance of the research at hand.

As part of the INNOVATE Research project, our team developed a youth engagement curriculum, available for download.^19^ It was built based on our reviews of the literature and our progressive experience engaging youth and bringing researchers onto projects with strong youth engagement components.^15^, ^17^ The curriculum addresses many of the concerns raised by researchers in the current findings, such as providing practical guidance on many of the steps involved in youth engagement, how to obtain funding and how to report on one's youth engagement activities in ways that will be valued by the research community. However, participants also indicated considerable interest in more active forms of training. The workshops and mentorship sessions delivered as part of the project addressed this additional need, by active training and providing on‐going mentorship, while taking the first steps towards building a network of youth‐engaged researchers by bringing youth‐engaged researchers together. The findings of the current study will be used to progressively refine our capacity‐building activities and youth engagement curriculum to best meet the needs of researchers aiming to expand and enhance their youth engagement research work.

Several limitations should be kept in mind when interpreting the findings. Notably, this is not a general sample of academics; rather, it is a sample specifically composed of individuals interested in building their capacity in youth engagement, which is reflected in the high levels of interest. As such, promoters of engagement will reach this primary audience first in their capacity development initiatives, and their needs are of primary importance. The data were also collected in self‐report format prior to registration for a workshop and may have been affected by social desirability. A larger sample size and more geographical diversity would also increase the generalizability of the findings, although the sample did span three large urban centres across Canada. The impact of the workshop is not assessed as part of the current manuscript. Furthermore, feedback from the youth who are ultimately engaged in the research was not collected; it is important to understand the barriers and facilitators from the engaged youth perspective to ensure that engagement learnings are applied in manners that translate into meaningful engagement experiences for youth as we move towards best practices in youth engagement. Also required is research specifically identifying the impacts of youth engagement on the evidence ultimately produced.

Engaging youth in research has substantial benefits for the feasibility and relevance of the work produced, from a youth‐focused and patient‐oriented research perspective. There is growing appreciation of this type of work among academics, institutions and funders, and a growing willingness among researchers to acquire training to engage youth appropriately. Concrete, active training opportunities and networks of youth‐engaged researchers are required, as are mechanisms to formally acknowledge the value of working in this way. Those promoting youth engagement in research are encouraged to consider these findings in their promotion and training endeavours.

## CONFLICT OF INTEREST

None.

## Data Availability

The data that support the findings of this study are available from the corresponding author upon reasonable request.
